# Effect of age of initiation of mammography breast cancer screening

**DOI:** 10.3332/ecancer.2024.1723

**Published:** 2024-06-28

**Authors:** Isabel Saffie-Vega, Sergio Muñoz-Navarro, Macarena Manríquez-Mimica, Jorge Sapunar-Zenteno

**Affiliations:** 1 Departamento de Investigación del Cáncer, Instituto Oncológico Fundación Arturo López Pérez, Unidad de Investigación Epidemiológica, Santiago 7500000, Chile; 2 Instituto Oncológico Fundación Arturo López Pérez, Unidad de Cirugía Oncológica de la Mama, Santiago 7500000, Chile; 3 Centro de Excelencia CIGES, Facultad de Medicina, Universidad de La Frontera, Temuco 4780000, Chile; a https://orcid.org/0000-0002-4723-5750; b https://orcid.org/0000-0003-0663-0575; c https://orcid.org/0009-0007-3540-1487; d https://orcid.org/0000-0001-9908-8054

**Keywords:** cancer screening, breast cancer, risk factors

## Abstract

**Introduction:**

Mammography is an excellent resource to reduce the burden of premature death associated with breast cancer; however, screening is only recommended between the ages of 50 and 69 years.

**General objective:**

To compare the frequency of suspicious and non-diagnostic mammograms for breast cancer when screening Chilean women between the ages of 40 and 50 years.

**Patients and methods:**

Cross-sectional study nested in a breast cancer screening programme in Chilean women >40 years old, conducted between 2017 and 2021. Demographic variables and risk factors are described. To establish the effect of age on screening, we calculated the number needed to screen for a Breast Imaging Reporting and Data Reporting System 4 or 5 mammogram when screening over 40 years or over 50 years.

**Results:**

We studied 137,690 women screened for breast cancer since the age of 40 years. The median age was 54 years (range 40–93 years). 64.7% of women were postmenopausal, 4.79% were nulliparous and 14% of post-menopausal women were receiving hormone replacement therapy. To find a suspicious mammogram, 170 women over 40 years and 149 women over 50 years would have to be screened.

**Conclusion:**

By changing the starting age of screening from 50 to 40 years 21 more women would have to be screened to detect a suspicious mammogram and if screened from age 50 and not from age 40 years 21% of total suspicious mammograms would remain unidentified.

## Introduction

Breast cancer screening by mammography remains one of the most controversial issues in contemporary health care. However, mammography screening is still the best strategy to reduce the burden of premature death associated with breast cancer [1]. Available evidence indicates that mammography screening in women aged 50–69 years reduces breast cancer mortality by 15%–21%. The effect on mortality in women younger than 50 years and older than 70 years is still debated [1, 2].

The incidence rate of breast cancer has surpassed that of lung cancer in women, with approximately 2.3 million new cases by 2020 and accounting for 11.7% of cancer incidence worldwide. It is the fifth leading cause of cancer mortality worldwide, with 685,000 deaths. Breast cancer incidence rates in high human development index (HDI) countries are 88% higher than those in medium and low HDI countries. In contrast, mortality rates in the latter are 17% higher [3]. These differences can be partly explained by early detection through mammographic screening [4, 5].

The Breast Imaging Reporting and Data Reporting System (BI-RADS) is an image classification system proposed by the American College of Radiology in 1986. The BI-RADS score was implemented to standardise risk assessment and quality control of mammography. The latest edition of BI-RADS (2013) includes six categories for findings, with an approximate risk of malignancy ranging from virtually zero to more than 95%. The report should be structured and include the following sections: breast density, imaging findings (using the appropriate lexicon), final assessment and management [6, 7].

In Chile, mammography has been included in public and private health systems by law since 2005. Coverage by age range and frequency has been increasing since then and currently, a free mammogram is guaranteed every 2–3 years for women aged 50–69 years [8]. However, it is estimated that only 40% of beneficiaries use the mammography offered by law [9]. Thus, despite the actions taken, there has been no impact on breast cancer mortality, which remains the leading cause of cancer death in Chilean women [10, 11].

Our institution is an oncology centre that offers mammograms through mobile clinics to women throughout Chile. This allows patients with limited resources or from remote areas of the country to have access to routine breast imaging. This paper shows the results of 137,690 mammograms performed using portable mammography between 2017 and 2021 in different communes of Chile.

## Materials and methods

Descriptive observational study, based on the database of a breast cancer screening programme for Chilean women between 2017 and 2021. This programme has been carried out since 2007, through agreements between the Donations and Charity Management of the Arturo López Pérez Foundation Oncology Institute (FALP) and municipalities and companies throughout the country. To select the subjects to be screened, the applicant institutions must apply the following criteria:

- Women over 40 years old (35 years old in case of family history and/or with a doctor's order)
- Not be pregnant or suspected to be pregnant.
- Not be breastfeeding.
- Not having had a mammogram in the last year.

FALP provides eight mobile clinics, equipped with Hologic SDA-SYS-3000-2D and LORAD 4-000-0029 mammograms, which travel to the agreed location for the screening. Along with the mammography, the medical technologist in charge applies a questionnaire that includes demographic and social variables and risk factors for breast cancer.

To conduct our study, we obtained an anonymised database of the screening programme between January 2017 and December 2021 from FALP's Donations and Charity Management. As we did not have access to the sensitive data of the screened subjects, we asked the Scientific Ethical Committee of FALP for a waiver of informed consent.

The analysis included women aged 40 years or older who had BIRADS categorisation in the database. The variables considered were age in years and by age strata in decades, age at last menstruation, postmenopausal status (defined as having had last menstrual period 1 year or more since the date of the examination), parity, cumulative lactation in months, use of hormonal therapy (contraceptives or substitution therapy), family history of breast cancer and mammography result (BIRADS classification). BIRADS categories 0 and 3 were grouped as non-diagnostic mammography because of the need for further examination and categories 4 and 5 as suspicious mammography.

To establish the external validity of the study, the distribution by age strata of the population analysed was compared with that of the 2017 census, using the goodness-of-fit model and the *χ* test^2^.

According to the nature of the variables, the results are described as frequencies or summary measures (mean or median) and dispersion (standard deviation or range). The frequency of suspicious mammograms (BIRADS 4-5) and non-diagnostic mammograms (BIRADS 0 and 3) was calculated for screening from the age of 40 years and from the age of 50 years. Alternatively, the number needed to screen for a suspected case was calculated. To assess association, ×2 was used for proportions with a *p*-value <0.05. Data analysis was performed with STATA 17.0 (StataCorp. 2021. Stata Statistical Software: Release 17. College Station, TX: StataCorp LLC).

## Results

After purging the database of cases with incomplete or inconsistent information, 137,690 women undergoing breast cancer screening between January 2017 and December 2021 were left for analysis. All of them are treated in Family Health Centres (CESFAM) and the costs are covered by the National Health Fund (FONASA). The median age was 54 years (40–94 years).

**Figure 1.**
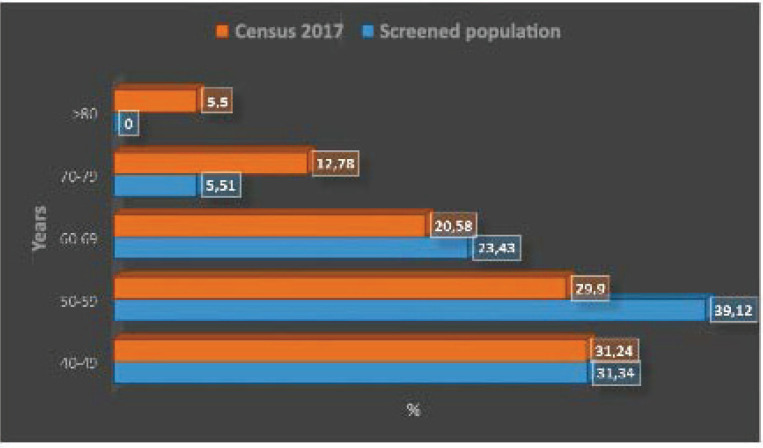
Age strata by decade in women aged 40 years and older in screened population versus Chile 2017 census population.

Figure 1 shows the distribution of the age strata by decade, with a predominance of the fifth and sixth decade of life. Figure 1 compares the relative importance of age strata by decade in the screened population and in the female population aged 40 years or older in the 2017 census, highlighting the lower relative importance of the strata 70–79 years and over 80 years in the screened population. The age distribution of the two populations differs significantly (*p* < 0.0001).

Table 1 presents some gynaecoobstetric variables associated with breast cancer risk, highlighting that only 4.79% of the screened women were nulliparous and that 14.4% were using or had used hormone replacement therapy. 19.43% had a family history of breast cancer and 8.22% had at least one first-degree relative with breast cancer.

Table 2 shows the relative importance of the BIRADS categories in women screened for breast cancer, with 25.32% of mammograms being non-diagnostic (BIRADS 0 and 3) and 0.59% suspicious for cancer (BIRADS 4-5).

**Figure 2.**
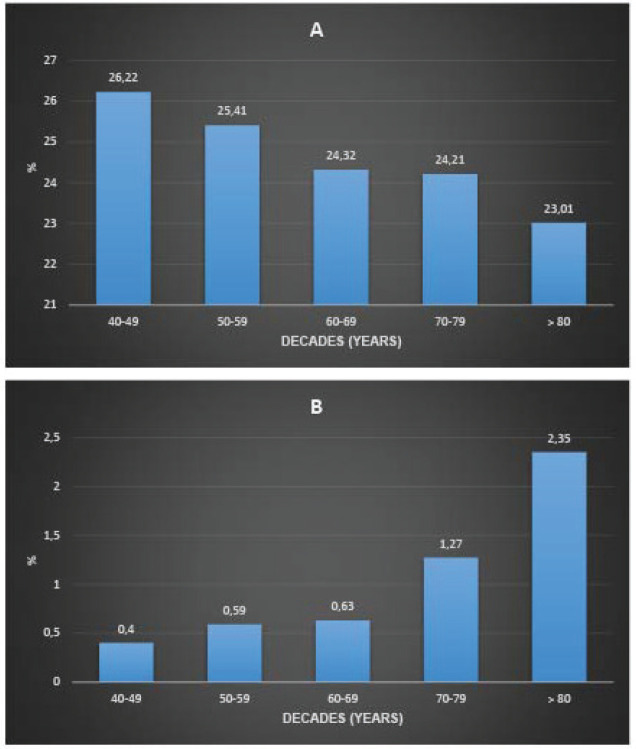
Proportion of non-diagnostic mammograms (a): suspicious mammograms (b): age stratum (Decennia) in 137,690 women screened with mammography between January 2017 and December 2021.

Figure 2 presents the proportion of non-diagnostic and suspicious mammograms by age group. There is a decreasing trend in the proportion of non-diagnostic mammograms and an increasing proportion of suspicious mammograms with age. The proportion of non-diagnostic mammograms was 26.53% in premenopausal women and 24.7% in post-menopausal women (*p*-value < 0.001).

In this population, when screening women from the age of 40, 0.59% had suspicious mammograms and 25.33% had non-diagnostic mammograms. In other words, 171 women from the age of 40 would have to be screened for a suspicious mammogram, accepting 43 non-diagnostic examinations. If women aged 50 were screened, the proportion of suspicious mammograms would be 0.67% and the proportion of non-diagnostic mammograms would be 24.9%. In other words, 149 women from the age of 50 would have to be screened to detect a suspicious mammogram, accepting 37 non-diagnostic examinations. On the other hand, not screening women under 50 would have meant not screening 171 suspicious mammograms (Figure 2).

Table 3 presents the effect of some variables on the risk of having a suspicious mammogram (BIRADS 4-5). A history of breast cancer in first-degree relatives and nulliparity stand out as risk factors with a significant effect on having suspicious mammograms. The lack of effect of hormone replacement therapy use is striking (OR 0.81 95% CI 0.585–1.119 *p*-value 0.2124).

## Discussion

The benefit of mammographic screening in the female population aged 50–69 years is indisputable, considering the high incidence of breast cancer and the reduction in mortality attributable to early diagnosis. However, 50% of breast cancers are diagnosed in women aged 65 years and older [12]. In the population studied, this age group was under-represented compared with the Chilean population pyramid from the 2017 census, and we postulate two reasons for this. Firstly, a large part of this age group is outside the range of free coverage guaranteed by the Chilean Ministry of Health (MINSAL), which could lead health personnel to exclude them, and also that women over 65 years of age often have problems with access or lack of education regarding the importance of breast cancer screening. That said, there is consensus that mammographic screening should not be based solely on age, but rather on each woman's comorbidities, functionality and life expectancy [13, 14].

Screening women between 40 and 50 years of age is also controversial, considering the technical difficulty due to the higher breast density [15] that reduces the diagnostic value of mammography and the lower incidence of mammographically detected tumours in this age range [16]. However, in the study cited above, a significant reduction in 10-year mortality was observed in the 40–50 age group when mammographic screening was performed (RR 0.75 95% CI 0.58–0.97 *p* = 0.029). According to our results, when screening women from the age of 40, we found 0.59% of suspicious mammograms (BI-RADS 4 or 5) and 25.33% of non-diagnostic mammograms (BI-RADS 0 and 3), which would require complementary examinations. When screening from the age of 50 in the same population, these figures are 0.67% and 24.9%, respectively. In other words, 171 women over 40 need to be screened to find a suspicious mammogram and 66 non-diagnostic mammograms need to be accepted. In the population over 50 years of age, 149 women need to be screened to find a suspicious mammogram and 37 non-diagnostic mammograms need to be accepted. Finally, screening women over 50 would have left 171 women between 40 and 50 years old with a suspicious test undiagnosed.

In our population, screening with mammography from the age of 40 would generate a 27% increase in demand for histological study, which in absolute terms is small (171 cases in 137,690 women screened).

Multiple risk factors for developing breast cancer have been studied [17, 18]. In our study, we found that a history of having first-degree relatives with breast cancer and nulliparity were associated with the finding of suspicious mammography (OR 1.35 and 1.4, respectively). Notably, these figures are consistent with international studies [17–19].

The limitations of our study include not having the histopathological report of those patients with suspicious mammography who underwent puncture for biopsy, which did not allow us to compare the proportion of false positives when screening from the age of 40 years and from the age of 50 years. The Donations and Charity Management of FALP did not participate in the selection of the women screened, and therefore, we do not know whether this was by spontaneous consultation or by referral by health personnel, which is relevant to establish the origin of the low adherence to the national screening programme in Chile. Finally, as we did not participate as researchers in the development of the questionnaire applied to the screened population, we were unable to assess the effect of other risk factors such as smoking, alcohol consumption or nutritional status.

## Conclusion

When screening with mammography in a population of women over 40 years of age, we found that the age stratum over 65 years is under-represented in relation to the 2017 Chilean census population. Including the stratum between 40 and 50 years of age results in a modest increase in the proportion of non-diagnostic mammograms and a non-significant reduction in the proportion of suspicious mammograms. Further studies are needed to expand the information collected in new studies to understand the effect of other risk factors, the reasons for screening, and especially the results of complementary examinations and histopathological study.

## Conflicts of interest

None of the authors declare any conflict of interest.

## Funding

The study was not funded.

## Author contributions

**Table:** 

Author	Conceptualisation	Methodology	Administration	Data analysis	Writing draft	Writing Edition
Isabel Saffie						
Sergio Muñoz						
Macarena Manriquez						
Jorge Sapunar						

## Figures and Tables

**Table 1.** Obstetric-gynecological variables associated with breast cancer risk in 137,690 women screened with mammography between January 2017 and December 2021.

Variable	Description *N* = 137.690
Age at Menarche (Average (SD))	13.9 (3.99) years
Menarche before age 9 (%)	0.43
Age at first gestation (Average (SD))	21.5 (5.04) years
Age at last menstrual period (Average (SD))	46.2 (9.51) years
Postmenopause (%)^a^	64.7
Parity (%) 0 1 2 3 4 5 6 >7	4.79 12.79 32.14 28.06 13.32 5.23 2.13 1.54
Cumulative lactation (%) < 6 meses 6–18 meses 18–36 meses >36 meses	15.3 36.88 26.96 20.86
Hormone replacement therapy (%)	14.40
Any family member with breast cancer (%) At least one first-degree relative with breast cancer (%)	19.43 7.74

a Defined as having last menstrual period 1 year or more before or after the date of the examination

**Table 2.** Relative importance of BI-RADS categories in 137,690 women screened with mammography between January 2017 and December 2021.

BI-RADS	Frequency	%
0	20.247	14.70
1	38.393	27.88
2	63.617	46.20
3	14.627	10.62
4	675	0.49
5	131	0.1
Total	137.690	100

**Table 3.** Effect of some variables on the risk of having a suspicious mammogram (BI-RADS 4 or 5).

Variable	OR	*p*-Value
First-degree relative (+)	1.352	0.0031
Hormone therapy use	0.818	0.2124
Nulliparity	1.402	0.0171
